# HIV among women in the WHO European Region – epidemiological trends and predictors of late diagnosis, 2009-2018

**DOI:** 10.2807/1560-7917.ES.2019.24.48.1900696

**Published:** 2019-11-28

**Authors:** Otilia Mårdh, Chantal Quinten, Giorgi Kuchukhidze, Nicole Seguy, Masoud Dara, Andrew J Amato-Gauci, Anastasia Pharris, on behalf of the ECDC/WHO HIV Surveillance network

**Affiliations:** 1 European Centre for Disease Prevention and Control (ECDC), Stockholm, Sweden; 2 WHO Regional Office for Europe, Copenhagen, Denmark; 3 The members of the ECDC/WHO HIV network are acknowledged at the end of the article

**Keywords:** HIV infection, Europe, women, epidemiology, late diagnosis, multivariate analysis, surveillance

## Abstract

Human immunodeficiency virus (HIV) transmission among women remains an issue in the WHO European Region, with nearly 50,000 women diagnosed in 2018 and over half (54%) diagnosed late. Although new HIV diagnoses declined between 2009 and 2018 in the West of the Region, they increased in the Centre and East. Understanding the characteristics of women diagnosed with HIV can inform gender-sensitive prevention services including pre-exposure prophylaxis and early testing and linkage to care.

Globally, more women are living with human immunodeficiency virus (HIV) than men [1]. However, in the World Health Organization (WHO) European Region, twice as many men are newly diagnosed with HIV than women each year [2]. Nevertheless, large numbers of HIV infections occur in women in Europe annually suggesting that further efforts toward prevention across the Region are warranted for countries to achieve Universal Health Coverage for all and meet the Sustainable Development Goal 3 target of ending AIDS by 2030 [3]. Here, we describe demographic and clinical characteristics and trends among women diagnosed with HIV in the WHO European Region and identify risk factors for late diagnosis by sub-Region to provide information for enhanced targeted prevention and testing.

## Data collection and analysis

All HIV diagnoses between 2009 and 2018, reported by the 53 countries in the WHO European Region were collected from the joint surveillance database of the European Centre for Disease Prevention and Control (ECDC) and WHO Regional Office for Europe.

Women were categorised as all people newly diagnosed with HIV with reported female sex, regardless of age. In the majority of European countries, the sex category is binary so it is unknown whether transwomen may be included in reported cases. Countries were grouped in three sub-regions based on geographic and broad epidemiological patterns [2]: (i) West (Andorra, Austria, Belgium, Denmark, Finland, France, Germany, Greece, Iceland, Ireland, Israel, Italy, Luxembourg, Malta, Monaco, Netherlands, Norway, Portugal, San Marino, Spain, Sweden, Switzerland, United Kingdom) (ii) Centre (Albania, Bosnia and Herzegovina, Bulgaria, Croatia, Cyprus, Czech Republic, Hungary, Montenegro, North Macedonia, Poland, Romania, Serbia, Slovakia, Slovenia, Turkey), and (iii) East (Armenia, Azerbaijan, Belarus, Estonia, Georgia, Kazakhstan, Kyrgyzstan, Latvia, Lithuania, Republic of Moldova, Russian Federation, Tajikistan, Turkmenistan, Ukraine, Uzbekistan). 

Migrant status was classified based on the reported country of birth or region of origin. Late diagnosis was defined as having a CD4+ T-cell count ≤ 350 cells/mm^3^ at HIV diagnosis.

All WHO European Region countries and territories except Tajikistan, Turkmenistan and Uzbekistan provided overall HIV rates by sex for the entire period 2009–2018. For countries and territories reporting case-based data for 2018 (all European countries and territories except for Russian Federation, Tajikistan, Turkmenistan and Uzbekistan), descriptive statistics were produced for age, HIV transmission and migrant status and compared across sub-regions. Time series analysis was performed to assess any statistically significant (p < 0.05) difference in trends (2009–2018) between men and women overall for the WHO European Region and at sub-region level. Multivariable logistic regression was performed for each sub-region separately for cases aged over 14 years to assess the association between late diagnosis and age, route of HIV transmission and migrant status. Records with unknown values for late diagnosis were omitted and the unknown values of age, transmission and migrant status were imputed using the ECDC HIV Estimates Accuracy Tool [4]. The association was assessed with the odds ratio (OR), its 95% confidence interval (CI) and the p value (α set at 5%) of the Wald χ^2^ statistic.

## New HIV diagnoses in women in the European Region, 2018

**Figure 1:**
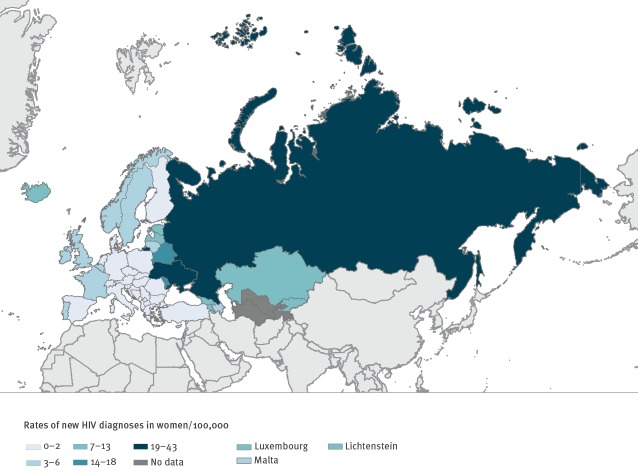
Rates of new HIV diagnoses in women per 100,000 population in the World Health Organization European Region, 2018 (n = 49,929)

In 2018, there were 49,929 new HIV diagnoses among women of all ages in the WHO European Region, representing about one-third (35.3%) of 141,552 new diagnoses that year. Of 49,929 new diagnoses in women, the majority (86%; n = 42,948) were in the East sub-region), followed by the West (12%; n = 6,000) and the Centre (2%; n = 981). Overall, there were 11.2 new HIV diagnoses per 100,000 women in the WHO European Region in 2018. Sub-regional rates varied from 33.2 per 100,000 women in the East to 2.8 in the West and 1.0 in the Centre; within each sub-region, the notification rates also varied (Figure 1).

HIV: human immunodeficiency virus.

Characteristics of women diagnosed with HIV in 2018 are presented in Table 1 for the 16,664 cases reported in case-based format. For the WHO European Region overall, the median age for women at diagnosis was 37 years (IQR: 30–46), with no statistically significant differences between sub-regions (p = 0.146). The largest proportion of women across all sub-regions were in the age group 30–49 years (60%; n = 9,928). In 2018, the large majority (92%; n = 13,240) of women in the WHO European Region were reported to have acquired HIV through heterosexual transmission, with only small variation between sub-regions. Transmission through injecting drug use accounted for 7% (n = 969) of all new HIV diagnoses among women, representing 9% (n = 827) of the reported cases in the East, 4% (n = 20) in the Centre and 3% (n = 122) in the West. At WHO European Region level in 2018, 75% (n = 11,630) of the HIV diagnoses were among native-born women, with the remainder of cases among migrant women originating from within the European Region (5%; n = 825) or from outside of Europe (19%; n = 2,989). Distribution by migration status differed across the sub-regions (Table 1).

**Table 1:** Characteristics of women diagnosed with HIV, World Health Organization European Region, 2018 (n = 16,664)

Characteristics	West		Centre		East		Total WHO European Region	
	n	%	n	%	n	%	n	%
**Age (years)(n = 16,652^a^)**								
< 15	74	1	14	1	101	1	189	1
15–19	156	3	24	2	133	1	313	2
20–24	451	8	79	8	583	6	1,113	7
25–29	828	14	142	14	1,216	13	2,186	13
30–39	1,918	32	338	34	3,523	36	5,779	35
40–49	1,393	23	242	24	2,514	26	4,149	25
50 +	1,167	19	153	15	1,603	17	2,923	17
**Total**	**5,987**	**100**	**992**	**100**	**9,673**	**100**	**16,652**	**100**
**Transmission mode (n = 14,444^b^)**								
Heterosexual contact	4,216	94	500	93	8,524	90	13,240	92
Injecting drug use	122	3	20	4	827	9	969	7
MTCT	90	2	14	3	84	1	188	1
Other (nosocomial, transfusion)	39	1	3	1	5	< 1	57	< 1
**Total**	**4,467**	**100**	**537**	**100**	**9,440**	**100**	**14,444**	**100**
**Migrant status (n = 15,444^c^)**								
Native	1,403	29	651	73	9576	99	11,630	75
European migrant	598	12	164	18	63	1	825	5
Non-European migrant	2,897	59	81	9	11	< 1	2,989	19
**Total**	**4,898**	**100**	**896**	**100**	**9650**	**100**	**15,444**	**100**
**CD4+ T-cell count at diagnosis (n = 11,984^d^)**								
350 +	1,711	47	145	43	3,667	46	5,523	46
≤ 350	1,903	53	194	57	4,364	54	6,461	54
**Total**	**3,614**	**100**	**339**	**100**	**8,031**	**100**	**11,984**	**100**

HIV: human immunodeficiency virus; MTCT: mother-to-child transmission; WHO: World Health Organization.

^a^ 12 cases from the West and 2 cases from the Centre with missing information on age not included.

^b^ 1,532 cases from the West, 457 cases from the Centre and 233 cases from the East with missing information on transmission not included.

^c^ 1,532 cases from the West, 457 from the Centre and 23 from the East with missing information on transmission not included.

^d^ 2,311 cases from the West, 641 from the Centre and 1,541 from the East with missing information on CD4+ T-cell count not included, a further 189 cases under 15 years of age not included due to different CD4+ T-cell count thresholds for late diagnosis.

Data include all countries that reported case-based data in 2018 (all countries in the WHO European region except Russian Federation, Tajikistan, Turkmenistan, and Uzbekistan).

## Late diagnosis

Information on CD4+ T-cell count among women aged over 14 years diagnosed with HIV in 2018 was available for 11,984 individuals; these were included in the risk factor analysis. The majority (54%) of women were diagnosed late (CD4+ T-cell count at HIV diagnosis <350 cells/mm^3^)(Table 1).

In multivariable analysis, older age was associated with an increased odds of late diagnosis in all sub-regions (Table 2). More specifically, in the West sub-region, the odds of being diagnosed late was double (OR: 2.02; 95% CI: 1.25–3.26) among women 25–29 years old and four times higher (OR: 4.13; 95% CI: 2.58–6.61) among women 50 years and older compared with younger women (15–19 years) (Table 2). Similarly, among women in the East sub-region, the odds of being diagnosed late increased with age (Table 2). For women in the Centre sub-region, being 40–49 years old, was the only age group at risk for late diagnosis (OR: 4.50; 95% CI: 1.13–17.97) compared with younger women (15–19 years). Transmission mode was associated with late diagnosis only among women in the East sub-region, where women who acquired HIV through injecting drug use were less likely to be diagnosed late than women in the East that acquired the infection though heterosexual transmission (OR: 0.72; 95% CI: 0.61–0.85). No association was found between migrant status and late diagnosis in any of the sub-regions (Table 2).

**Table 2:** Risk factors for late diagnosis in women newly diagnosed with HIV infection, WHO European sub-region, 2018 (n = 11, 984)

Risk factors (independent variables)	WHO European sub-region									
	West n = 3,614^a^			Centre n = 339^b^				East n = 8,031^c^		
	OR	95% CI	P value	OR	95% CI		P value	OR	95% CI	P value
**Age (years)**										
15–19	Ref			Ref				Ref		
20–24	1.18	0.71–1.96	0.525	0.82		0.19–3.59	0.791	1.08	0.69–1.68	0.737
25–29	2.02	1.25–3.26	**0.004**	1.65		0.42–6.49	0.477	1.88	1.24–2.86	**< 0.001**
30–39	2.60	1.64–4.15	**< 0.001**	2.51		0.66–9.55	0.177	2.91	1.93–4.38	**< 0.001**
40–49	3.17	1.98–5.08	**< 0.001**	4.50		1.13–17.97	**0.033**	4.35	2.88–6.56	**< 0.001**
50 +	4.13	2.58–6.61	**< 0.001**	3.05		0.78–12.01	0.111	5.62	3.70–8.53	**< 0.001**
**HIV transmission**										
Heterosexual contact	Ref			Ref				Ref		
Injecting drug use	0.65	0.42–1.02	0.062	2.78		0.77–9.98	0.117	0.72	0.61– 0.85	**< 0.001**
MTCT	0.97	0.38–2.49	0.952	NC			NC	NC		
Other (nosocomial, transfusion)	1.39	0.07–27.61	0.684	NC			NC	NC		
**Migrant status**										
Native	Ref			Ref				Ref		
European migrant	1.24	0.69–2.23	0.318	1.37		0.69–2.72	0.361	1.23	0.91–1.66	0.182
Non-European migrant	1.19	1.00–1.43	0.052	1.65		0.52–5.28	0.394	0.64	0.11–3.81	0.629

CI: confidence interval; HIV: human immunodeficiency virus; NC: Not calculated (too small number); MTCT: mother-to-child transmission; Ref: reference.

^a^ 53% of women were diagnosed late.

^b^ 57% of women were diagnosed late.

^c^ 54% of women were diagnosed late.

P-value of the Wald χ^2^ statistic set at 0.05, statistically significant values in bold. For the independent variables (age, transmission mode and migration status), the unknown values were imputed using the ECDC HIV Estimates Accuracy Tool [4].

## Trends of HIV notifications, 2009–2018

**Figure 2:**
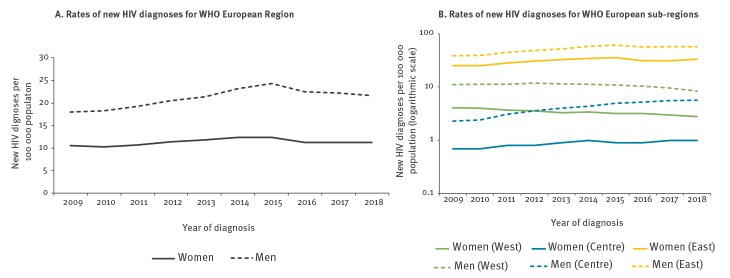
Rates of new HIV diagnoses by sex, year of diagnosis for World Health Organization European Region and sub-regions, 2009–2018

The notification rate of HIV diagnoses among women increased slightly (by 7%), from 10.5 per 100,000 women in 2009 to 11.2 in 2018 (Figure 2a). There were differences between sub-regions, with a 32% decrease in the notification rate among women in the West, in contrast with an increase of 43% in the Centre and 31% in the East (Figure 2b). A similar geographical pattern was observed among men (Figure 2b). No statistically significant (p > 0.5) differences in notification rates over time were reported between males and females within the WHO European Region or within each sub-region.

HIV: human immunodeficiency virus.

**Figure 3:**
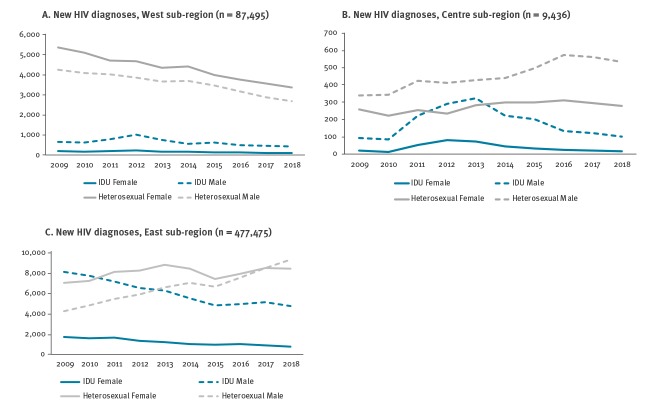
New HIV diagnoses by sex and transmission route, West, Centre and East sub-regions, 2009–2018

HIV: human immunodeficiency virus; IDU: injecting drug use.

In the West sub-region, new HIV diagnoses due to heterosexual transmission and injecting drug use declined between 2009 and 2018 among both men and women (Figure 3a). New diagnoses due to heterosexual transmission in the Centre sub-region increased substantially in men, while increasing only very slightly in women (Figure 3b). In the East sub-region, new HIV diagnoses attributed to injecting drug use declined in both men and women, with an increase in both sexes in infections attributed to heterosexual transmission (Figure 3c).

In panel A: Data from Italy and Spain were excluded due to changes in the coverage of surveillance over the period. In panel B: Data from Turkey and Poland were excluded due to incomplete data on transmission over the period. In panel C: Data from Russian Federation, Tajikistan, Turkmenistan and Uzbekistan were excluded due to incomplete data on transmission or incomplete reporting during the period.

**Figure 4:**
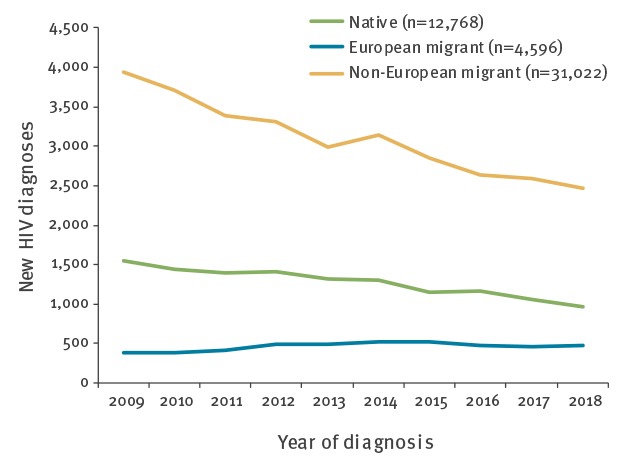
HIV diagnoses among women, by year of diagnosis and migration status, West sub-region, 2009–2018 (n = 46,386)

In the West sub-region, where migrant women represent a considerable proportion of HIV cases, new HIV diagnoses declined overall during 2009–2018 among non-European migrant women and increased slightly among migrant women from within Europe (Figure 4). New HIV diagnoses among native women declined.

HIV: human immunodeficiency virus.

Italy and Spain are excluded from trends reported here due to changes in the coverage of surveillance over the period.

## Discussion

In 2018, nearly 50,000 women were newly diagnosed with HIV, comprising one-third of the new diagnoses in the WHO European Region. The majority (86%) of the women diagnosed with HIV in 2018 were in the East of the Region and 54% were diagnosed late. Heterosexual transmission was the most common route of infection in women, the majority of those newly diagnosed were native to the reporting country (75% of all cases), and the age group most affected was 30–49 years (60% of all cases). This is in contrast with other sexually transmitted infections such as chlamydia and gonorrhoea where incidence is highest in women aged 15 to 24 [5,6].

There were differences in trends of HIV diagnoses during 2009–2018 across the sub-regions. While new HIV diagnoses among women have declined in the West sub-region during the last decade, particularly among non-European migrant women, they have increased in the Centre and East sub-regions, largely due to increased reports of heterosexual transmission among women. While risk factors for HIV acquisition among women are multi-faceted and complex, increasing incidence among male populations in the Centre and East sub-regions, combined with large populations living in the Centre and East with transmissible HIV due to low treatment coverage and viral suppression are likely factors impacting women in these sub-regions [7].

Behavioural and other risk factor information is limited within HIV surveillance systems on the European level. Evidence from the literature indicates that risk factors for women are often linked to risk factors of their male sexual partners, including: (i) history of injecting drug use, (ii) sex with men, (iii) hazardous alcohol drinking, or (iv) a sexual partner who originates from an area of high HIV prevalence [8,9]. Beyond partner risk factors, women with high numbers of unprotected sexual or injecting contacts are at higher risk for HIV acquisition, including female sex workers. Collection of more detailed information on socio-demographic, behaviour and partner characteristics for the women newly diagnosed with HIV would facilitate a better characterisation of the women most vulnerable to HIV acquisition and inform prevention polices, including better targeting of women most likely to benefit from pre-exposure prophylaxis (PrEP) for HIV [9]. In October 2019, Women Against Viruses in Europe (WAVE) within the European AIDS Clinical Society, took steps to collect such data via a survey for PrEP availability and implementation across the region, which, once published, could be inform policy makers in terms of prevention of new infections in women in Europe [10].

Late diagnosis among women is pervasive in all sub-regions. While evidence shows that testing within health services (including sexual health services and primary care), as well as partner notification and indicator condition testing are effective interventions and increase early diagnosis, their coverage is still limited in many European countries [11,12]. Enhanced strategies to better serve women for HIV testing in all settings, including, but not limited to antenatal screening, is essential. Given the median age of 37 years at HIV diagnosis, it is important that women in older age groups be offered sexual health counselling and HIV testing in a variety of settings in addition to antenatal screening. Vulnerable women in key population groups could be better reached for HIV testing through community-based services, however, in many settings these primarily provide services to men having sex with men. A positive association was found between an older age and late diagnosis. While this can be partially explained by a faster CD4+ T-cell depletion in individuals of older age [13], being older than 50 years was associated with late diagnosis in a Dutch cohort [14] and with increased risk behaviour among older women who inject drugs in Russia [15].

While data presented here represent HIV diagnoses rather than HIV incidence, they provide the most comprehensive picture to-date of the situation of women diagnosed with HIV in the European region. Women still comprise a sizeable population of those affected with HIV in Europe and counselling and prevention among them, combined with enhanced gender-sensitive HIV testing and linkage to care, is key in order for European countries to accelerate their progress towards achieving Universal Health Coverage and reaching the Sustainable Development Goal on ending AIDS by 2030.
